# Prevalence and Antibiotic Resistance Profile of *Clostridium perfringens* Isolated from Pork and Chicken Meat in Vietnam

**DOI:** 10.3390/pathogens13050400

**Published:** 2024-05-10

**Authors:** Hoang Minh Duc, Tran Thi Khanh Hoa, Cam Thi Thu Ha, Le Van Hung, Nguyen Van Thang, Hoang Minh Son, Gary A. Flory

**Affiliations:** 1 Department of Veterinary Public Health, Faculty of Veterinary Medicine, Vietnam National University of Agriculture Trau Quy, Gia Lam, Hanoi 12400, Vietnam; 2 Veterinary Hospital, Faculty of Veterinary Medicine, Vietnam National University of Agriculture Trau Quy, Gia Lam, Hanoi 12400, Vietnam; 3 Department of Anatomy and Histology, Faculty of Veterinary Medicine, Vietnam National University of Agriculture Trau Quy, Gia Lam, Hanoi 12400, Vietnam; 4 G.A. Flory Consulting, Mt. Crawford, VA 22841, USA; garyaflory@gmail.com

**Keywords:** *Clostridium* *perfringens*, chicken meat, pork, food poisoning, antimicrobial resistance

## Abstract

*Clostridium perfringens* is one of the most important zoonotic pathogens as it can cause food poisoning in humans and necrotic enteritis in both animals and humans. Meat, especially pork and chicken meat, is considered the main vehicle for the transmission of *C. perfringens* from animals to humans. The purpose of this study was to determine the prevalence, toxinotype, and antimicrobial resistance profile of *C. perfringens* isolated from pork and chicken meat sold in Vietnam. The isolation results showed that 15/50 (30%) of pork samples and 8/50 (16%) of chicken meat samples were contaminated with *C. perfringens*. The isolates exhibited their highest resistance rate to tetracycline (21/23; 91.30%) and clindamycin (10/23; 43.48%). On the contrary, their lowest resistance rates were observed in response to imipenem (2/23; 8.70%) and cefoxitin (1/23; 4.35%). In particular, 34.78% (8/23) of *C. perfringens* isolates were identified to be multidrug-resistant strains. The results of toxin genotyping indicated that all isolates were positive for the *cpa* gene and belonged to type A.

## 1. Introduction

*Clostridium perfringens* is a Gram-positive, anaerobic, non-motile, spore-forming pathogen commonly found in soil, sewage, food, and the gastrointestinal tract of warm-blooded animals, including humans [1,2,3]. This bacterium has been recognized as a public health concern as it can cause numerous diseases in humans and animals, including food poisoning [4,5,6]. In the United States, *C. perfringens* has been recognized as the second most common bacterial cause of foodborne infection, causing one million illnesses annually. From 1998 to 2010, 289 outbreaks of *C. perfringens* infections were confirmed, resulting in 15,208 illnesses, 83 hospitalizations, and 8 deaths [7]. In Europe, *C. perfringens* is the fourth most common bacterial cause of foodborne illness, infecting over 1500 people per year [3]. In Japan, *C. perfrigens* was responsible for about 20 to 40 outbreaks of food-borne diseases from 2000 to 2005 and approximately 4000 illness cases each year [8]. In other countries (Australia, England, and Wales), *C. perfringens* is also counted among the main causes of bacterial foodborne outbreaks [9,10]. Outbreaks of *C. perfringens* infections are often linked to the consumption of contaminated meat, particularly chicken meat [11]. 

*C. perfringens* is capable of producing 20 different toxins and extracellular enzymes [12]. Based on the production of six major toxins, Alpha (CPA), Beta (CPB), Epsilon (ETX), Iota (ETX), Enterotoxin (CPE), and necrotic enteritis-β-like toxin (NetB), this bacterium is categorized into seven toxinotypes (A to G) [2,13]. All seven toxigenic types of *C. perfringens* produce CPA. Type A produces only CPA, while type B produces two additional toxins, including CPB and ETX. Types C, D, E, F, and G have been found to produce another single toxin in addition to CPA, namely CPB, ETX, ITX, CPE, or NetB, respectively [2,14]. Each specific toxinotype is known to be associated with certain diseases. For example, *C. perfringens* type A is frequently responsible for clostridial myonecrosis or gas gangrene and food poisoning in humans, but it is also associated with enterocolitis in pigs and horses or necrotic enteritis in chickens [13,15]. Type B is involved in dysentery, enteritis, and enterotoxemia in animals. Type C is a causative agent of necrotic enteritis in humans and animals. Type D is linked to pulpy kidney disease in humans and type E is known to cause enteritis in animals [15]. Type F is responsible for food poisoning in humans [15]. Type G is considered the main cause of necrotic enteritis in poultry [16] Therefore, the toxin typing of isolated *C. perfringens* strains is necessary to determine the potential hazard of these isolates and to identify the source of contamination across different steps of food production. 

Antibiotics are known to be the most effective means for treating bacterial infections; however, they are losing their efficacy due to the rise of antibiotic-resistant strains [17]. The overuse and misuse of antibiotics have been attributed to the development of antibiotic resistance [18,19]. In Vietnam, antibiotics have been used in livestock for various purposes such as disease prevention, treatment, and growth promotion [20,21]. As a result, Vietnam has been classified by the WHO as one of the countries with the highest level of antibiotic resistance [22]. However, studies on antibiotic resistance in Vietnam are limited and just focus only on a few pathogens such as *Escherichia coli* and *Salmonella* [23,24]. Reports on the antibiotic resistance profile of *C. perfringens* in Vietnam are scarce. The aim of this study is to (1) investigate the prevalence and toxigenic type of the *C. perfringens* isolated from pork and chicken meat sold in Hanoi, Vietnam, and (2) determine the antibiotic resistance profile of these *C. perfringens* isolates.

## 2. Materials and Methods

### 2.1. Sample Collection

A total of 100 raw meat samples (50 pork and 50 chicken meat; 100 grams/sample) were obtained from various retail stores in Gia Lam district, Hanoi city, Vietnam, from 2022 to 2023. All collected samples were stored in an ice box and brought back to the laboratory within 24 hours for the isolation of *C. perfringens*.

### 2.2. Isolation and Identification of C. perfringens from Pork and Chicken Meat

The isolation and identification of *C. perfringens* from pork and chicken meat samples were carried out according to the previously described method of the American Public Health Association (APHA) [25]. Briefly, a sample (25 g) was added to a bag (Seward Ltd, Worthing, UK) containing 225 mL of 0.1% Peptone Water (Oxoid Ltd, Hants, UK), homogenized by a Seward stomacher 400 circulator (Seward Ltd, Worthing, UK), and incubated for 24 h under anaerobic conditions at 37 °C. After incubation, the homogenate was serially diluted and 1 mL of an appropriate dilution was mixed with soft tryptose sulfite cycloserine agar (Oxoid Ltd, Hants, UK), supplemented with 5% egg yolk and perfringens selective supplement, and then poured on a Petri dish. After solidification, the plate was overlayed with a second TSC layer and incubated anaerobically for 24 h at 37 °C. Colonies with a black color and white halo were considered presumptive *C. perfringens*. Well-separated colonies were picked up for biochemical testing using an API 20A kit (Biome’rieux, Marcy I’ Etoile, France). For further confirmation, up to five single colonies per each plate were selected to detect the species-specific *16S-rRNA* gene using the method previously described by Tonooka et al. (2005) [26]. All *C. perfringens* strains were stored at −86 ˚C for further experiments.

### 2.3. Detection of Toxin Genes of C. perfringens Isolated from Pork and Chicken Meat

The toxin genes (*cpa, cpb, etx, iap, cpe)* of isolated *C. perfringens* strains were detected by multiplex PCR following the previous method described by [27], while the *netB* gene was detected by a simple PCR assay [28]. For DNA extraction, *C. perfringens* isolates were grown in 5 mL of brain heart infusion (BHI, Oxoid, Hants, UK) and incubated under anaerobic conditions at 37 °C for 24 h. The bacterial culture was then used for DNA extraction using a GeneJet Genomic DNA purification kit (Thermoscientific, Vilnius, Lithuania) according to the instruction of the manufacturer. The extracted DNA was stored at −20 °C until used for PCR reactions. The primers used in this study for the detection of the toxin genes of *C. perfringens* isolates are shown in Table 1.

A multiplex PCR reaction was performed in a total reaction volume of 25 µL containing 2.5 μL of 10 × PCR Buffer, 10 µL of 1 mM dNTPs, 5 µL of 1U Taq polymerase, 0.25 µL of 25 μM of each primer, 2μL of DNA template, and 2.5 µL of deionized water. A thermal cycling machine (Biorad T100, BioRad Laboratories, Hercules, CA, USA) was used to carry out a PCR amplification program consisting of initial denaturation at 94 °C for 2 min, followed by 34 cycles at 94 °C for 1 min, 55 °C for 1 min, and 72 °C for 1 min, and a final elongation at 72 °C for 10 min. The amplified PCR product was separated by electrophoresis on a 2% agarose gel and visualized under ultraviolet light by a BioRad Molecular Imager® GelDocTM XR (BioRad Laboratories, Hercules, CA, USA).

The amplification of the *netB* gene was performed on a 25 µL reaction volume comprising 2.5 μL of 10 × PCR Buffer, 5 µL of 1 mM dNTPs, 5 µL of 1U Taq polymerase, 1 µL of 5 μM of each primer, 2μL of DNA template, and 8.5 µL of deionized water. The amplification program is composed of an initial denaturation at 94 °C for 2 min, 35 cycles at 94 °C for 30 s, 55 °C for 30 s, and 72 °C for 1 min, followed by a final extension at 72 °C for 12 min. The PCR product was analyzed according to the method mentioned above. 

### 2.4. Antimicrobial Susceptibility of C. perfringens Isolates

The antimicrobial susceptibility of isolated *C. perfringens* strains was tested using the agar dilution method, following the guidelines of the Clinical and Laboratory Standards Institute (CLSI) [29]. The lowest concentration of an antimicrobial that inhibited the isolates’ growth visibly on Brucella agar supplemented with hemin (5 µg/mL), vitamin K1 (1 µg/mL), and laked sheep blood (5%, *v*/*v*), after 48 h of incubation at 37 °C, was defined as the minimum inhibitory concentration (MIC). A total of 7 antibiotics from 4 classes (Beta-lactams, Tetracyclines, Phenicols, and Lincosamides) were selected for this test, including ampicillin, cefoxitin, cefotaxime, imipenem, tetracycline, chloramphenicol, and clindamycin. The quality control strain used for the test was *Clostridium difficile* ATCC 700057 [29]. Isolates that showed a resistance to at least 1 antibiotic from 3 or more antibiotic classes were identified as multidrug-resistant strains.

## 3. Results

### 3.1. The Isolation and Identification of C. perfringens from Pork and Chicken Meat

A total of 23 (23%) *C. perfringens* strains were isolated from 50 pork and 50 chicken meat samples. The occurrence of *C. perfringens* in pork and chicken meat samples collected from traditional markets in Hanoi, Vietnam, were 15/50 (30%) and 8/50 (16%), respectively, indicating that the higher prevalence of *C. perfringens* in pork than chicken meat. 

### 3.2. Detection of the Toxin Genes of C. perfringens Isolates

**Figure 1:**
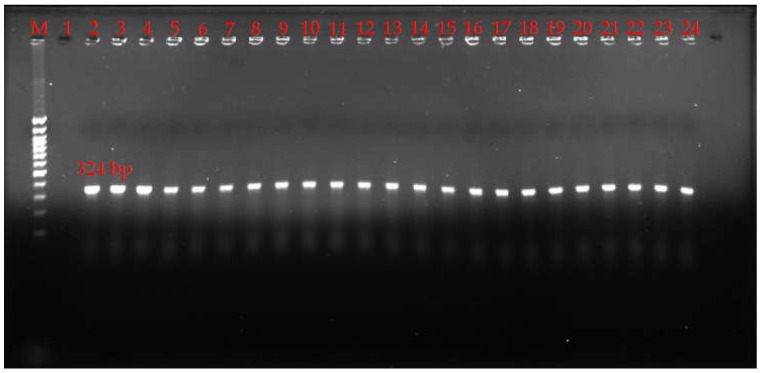
Multiplex PCR detecting toxin genes of *C. perfringens*. M: marker; Lane 1: negative control; Lane 2–24: isolated *C. perfringens* strains.

A multiplex PCR was used to classify the *C. perfringens* isolates into seven toxinotypes (A-G). The result revealed that all 23 isolated strains harbored only the *cpa* gene (Figure 1), indicating that they all belong to type A. 

### 3.3. Antimicrobial Resistance Profile of C. perfringens Isolated from Pork and Chicken Meat

Seven antibiotics from four classes were used for the antibiotic resistance test of *C. perfringens*. The results in Table 2 showed that the highest antibiotic resistance rate of *C. perfringens* isolates was to tetracycline (21/23; 91.30%), followed by clindamycin (10/23; 43.48%), ampicillin (8/23; 34.78%), chloramphenicol (7/23; 30.43%), and cefotaxime (5/23; 21.74%). In contrast, the lowest antibiotic resistance rates were observed with imipenem (2/23; 8.70%) and cefoxitin (1/23; 4.35%). 

The phenotypic antibiotic resistance of the isolated *C. perfringens* strains is shown in Table 3 and Table 4. All isolates were found to be resistant to at least one antibiotic class. A total of 11 antibiotic resistance patterns were detected. Among them, tetracycline resistance (TET) was the most common pattern, accounting for 43.48% (10/23) of strains, followed by TET-CHL, AMP-CTX-TET-CLI, and AMP-TET-CHL-CLI, with the same rate of 8.7% (2/23). The findings in Table 3 and Table 4 also showed that eight (34.78%) *C. perfringens* isolates were resistant to at least three antibiotic classes and identified as multidrug-resistant strains (MDR). 

## 4. Discussion

*C. perfringens* has been recognized as one of the most important foodborne pathogens [30]. The bacterium can produce many dangerous toxins including enterotoxin, which can cause food poisoning [31,32,33]. In addition, *C. perfringens* is capable of forming spores that allow this pathogen to survive under stressful conditions, such as high temperatures and aerobic environments, and eventually grow to such an extent that it can cause food poisoning [34,35,36]. *C. perfringens* is also known as a causative of necrotic enteritis in poultry and pigs. In this case, the bacterium infects, colonizes, and damages the intestinal tract of animals. During slaughter, *C. perfringens* may escape from the intestinal tract and contaminate the meat. Therefore, meat, especially pork and chicken, is considered to be one of the main vehicles for the transmission of *C. perfringens* from chickens and pigs to humans [37,38,39,40,41]. 

To date, studies on the occurrence and antimicrobial resistance of *C. perfrigens* isolates have mainly focused on humans and food-producing animals [42,43,44,45]. There are only a few publications on *C. perfringens* of food origin [46,47]. To the best of our knowledge, this is the first report on the prevalence and antimicrobial resistance profile of *C. perfringens* isolated from pork and chicken meat in Vietnam. In our study, a total of 23 (23%) out of 100 meat samples were positive for *C. perfringens*. The prevalence of *C. perfringens* in meat found in the present study was relatively lower than in some previous studies. For example, a study conducted by Wen and McClane showed that the prevalence of *C. perfringens* in retail pork and chicken meat was 38% and 27%, respectively [11]. Another study found that the prevalence of *C. perfringens* in chicken meat was 31% [48]. Jang et al. reported that 33% of retail chicken meat in Korea was positive for *C. perfringens* [47]. The findings of this study also showed that the rate of *C. perfringens* contamination in pork samples (30%, 15/50) was higher than in chicken meat samples (16%, 8/50), suggesting that the consumption of pork may lead to a higher likelihood of *C. perfringens* infection that chicken meat. On the contrary, a study in Korea reported that the highest prevalence of *C. perfringens* was recorded in chicken meat (33%, 33/100), followed by beef (5%, 5/50), while all pork samples (50) were negative for *C. perfringens*. The occurrence of *C. perfringens* in pork has been previously reported in Korea (5.0%) [49] and India (5.8 %) [50]. The different disinfection conditions during the handling, processing, and distribution of meat may influence these differences in the prevalence of *C. perfringens* [51]. In addition, sample size, sample type, sampling season, isolation techniques, and location can also affect the prevalence of *C. perfringens* [52]. 

*C. perfringens* type A has been previously reported to be the most common toxigenic type of bacterium associated with food poisoning in the United States, Europe, and Japan [8,53]. The results of our study are in line with previous studies, showing that all *C. perfringens* strains were identified as type A. Similar results were obtained in a study conducted by Zhang et al. in China, according to which the majority of their 168 *C. perfringens* isolates belonged to type A [54]. In Turkey, type A was also the only toxinotype found in turkey meat samples [53]. In Belgium, 71 *C. perfringens* strains isolated from broiler caeca were all categorized as type A [55]. In addition, a study performed in Korea revealed that the *C. perfringens* strains recovered from chicken meat and beef were all type A [47]. The opposite results were observed in a study conducted in Iran, which reported that type C was the most common type of strain in broiler meat samples [56]. In this study, all *C. perfringens* isolates carried only the *cpa* gene. It has previously been suggested that this gene may be universal in *C. perfringens* isolates of a meat origin [2]. The reason for the high prevalence of the *cpa* gene could be that the gene is located on the chromosome of *C. perfringens*. In contrast, its other toxin genes, *cpb*, *etx* and *itx,* are in plasmids, while *cpe* encoded for enterotoxin can be detected on either the plasmids or the chromosome [13]. A plasmid containing toxin genes is a mobile element and can be lost. This could explain the absence of other toxin genes in the *C. perfringens* strains isolated in this study. In addition, previous studies have already shown that the *cpe* gene is rarely detected in type A and that only 1–5% of *C. perfringens* isolates carried the *cpe* gene [8,11,57]. Although only *cpe*-negative type A was detected, the meat contaminated with *C. perfringens* isolates in this study was still risky to consume, as a previous study recently reported that *cpe*-negative type A *C. perfringens* can cause septicemia with intravascular hemolysis, with a mortality rate of 80% [58]. 

In Vietnam, veterinarians and even farmers have been using antibiotics for decades to prevent and treat animal diseases [59]. In the chicken and swine industries, a large amount of antibiotics is used for prevention without specific diagnoses [60]. It was also reported that about 43.7% of commercial animal feed sold in Vietnam contained at least one antibiotic [61]. Necrotic enteritis (NE), caused by *C. perfringens,* is one of the most important diseases in the poultry and swine industries. The use of antibiotics at low concentrations for prophylactic prevention is considered the most effective measure to control NE [62]. However, this consequently led to the emergence of antibiotic-resistant *C. perfringens* clones [62]. The results of our study showed that the *C. perfringens* from meat were highly resistant to tetracycline (21/23; 91.30%) and clindamycin (10/23; 43.48%). These findings are not surprising as these antibiotics have been widely used in many countries, including Vietnam, for the prevention of NE and other diseases in chickens and pigs [20,62]. Our results are in agreement with previous studies showing a high resistance rate of *C. perfringens* to tetracycline and clindamycin. In a study conducted by Beres et al. in Romania, it was reported that the resistance rate of *C. perfringens* isolated from food-producing animals to tetracycline was 71.4% [63]. A study performed by Jang et al. in Korea found that 100% of *C. perfringens* isolated from meat were tetracycline-resistant strains [47]. A study in Belgium obtained similar results and reported that the resistance rate of *C. perfringens* from broilers to tetracycline was 66.6% [55]. Another study in China has shown that 61.1% and 72.2% of meat-derived C. *perfringens* isolates were resistant to tetracycline and clindamycin, respectively [54]. The lowest resistance rates to tetracycline and clindamycin were also observed in a study conducted in Canada. *C. perfringes* isolated from poultry in that study were 50% and 40% resistant to tetracycline and clindamycin, respectively [42]. Our study also revealed that the resistance rate of *C. perfringens* to cefoxitin (4.35%) and imipenem (8.7%) was still low. This could be due to the fact that these antibiotics have not been commonly used in livestock, especially to prevent and treat the diseases caused by *C. perfringens*. Similar results were also found in earlier studies [64,65]. 

## 5. Conclusions

The emergence of antimicrobial resistance in foodborne pathogens has become a global problem. Our study is the first report on the prevalence and antibiotic resistance profile of *C. perfringens* isolated from meat in Vietnam. The results showed that pork and chicken meat was contaminated with *cpe*-negative *C. perfringens* type A. The *C. perfringens* isolates in this study exhibited their highest resistance rate to tetracycline and clindamycin. A large proportion of *C. perfringens* isolates were identified as multidrug-resistant strains, indicating a potential risk to human health.

## Figures and Tables

**Table 1:** Primers for the detection of the *16S-rRNA* gene and toxin genes of *C. perfringens* isolated from pork and chicken meat.

Target Gene	Primer Name	Primer Sequence (5′–3′)	Amplicon Size (bp)	Reference
*16S-rRNA*	16S-F	TAACCTGCCTCATAGAGT	481	[26]
	16S-R	TTTCACATCCCACTTAATC		
*cpa*	cpa-F	GCTAATGTTACTGCCGTTGA	324	[27]
	cpa-R	CCTCTGATACATCGTGTAAG		
*cpb*	cpb-F	GCGAATATGCTGAATCATCTA	196	
	cpb-R	GCAGGAACATTAGTATATCTTC		
*cpb2*	cpb2-F	AGATTTTAAATATGATCCTAACC	567	
	cpb2-R	CAATACCCTTCACCAAATACTC		
*cpe*	cpe-F	GGAGATGGTTGGATATTAGG	233	
	cpe-R	GGACCAGCAGTTGTAGATA		
*etx*	etx-F	GCGGTGATATCCATCTATTC	655	
	etx-R	CCACTTACTTGTCCTACTAAC		
*iap*	iap-F	ACTACTCTCAGACAAGACAG	446	
	iap-R	CTTTCCTTCTATTACTATACG		
*netB*	NetB-F	GCTGGTGCTGGAATAAATGC	384	[28]
	NetB-R	TCGCCATTGAGTAGTTTCCC		

**Table 2:** Antimicrobial resistance profile of *C. perfringens* isolated from pork and chicken meat.

Antibiotic Class		Antibiotic Agent	No. *C. perfringen* Isolates (%)		
			Chicken Meat (n = 8)	Pork (n = 15)	Total (n = 23)
Beta-lactams	Penicillins	ampicillin (AMP)	3 (37.5)	5 (33.33)	8 (34.78)
	Cephalosporins	cefoxitin (FOX)	0 (0)	1 (6.67)	1 (4.35)
		cefotaxime (CTX)	2 (25)	3 (20)	5 (21.74)
	Carbapenem	imipenem (IPM)	0 (0)	2 (13.33)	2 (8.7)
Tetracyclines		tetracycline (TET)	8 (100)	13 (86.67)	21 (91.3)
Phenicols		chloramphenicol (CHL)	2 (25)	5 (33.33)	7 (30.43)
Lincosamides		clindamycin (CLI)	4 (50)	6 (40)	10 (43.48)

**Table 3:** Antibiotic resistance patterns of *C. perfringens* isolated from pork and chicken meat.

No. of Antibiotics	Resistance Pattern	No. *C. perfringen* Isolates (%)		
		Chicken Meat (n = 8)	Pork (n = 15)	Total (n = 23)
1	TET	4 (50)	6 (40)	10 (43.48)
	CLI	0 (0)	1 (6.67)	1 (4.35)
2	TET-CLI	1 (12.5)	0 (0)	1 (4.35)
	CHL-CLI	0 (0)	1 (6.67)	1 (4.35)
	TET-CHL	0 (0)	2 (13.33)	2 (8.7)
3	AMP-TET-CLI	0 (0)	1 (6.67)	1 (4.35)
4	AMP-CTX-TET-CLI	1 (12.5)	1 (6.67)	2 (8.7)
	AMP-TET-CHL-CLI	1 (12.5)	1 (6.67)	2 (8.7)
5	AMP-CTX-TET-CHL-CLI	1 (12.5)	0 (0)	1 (4.35)
	AMP-FOX-CTX-IPM-TET	0 (0)	1 (6.67)	1 (4.35)
6	AMP-CTX-IPM-TET-CHL-CLI	0 (0)	1 (6.67)	1 (4.35)

AMP, ampicillin; FOX, cefoxitin; CTX, cefotaxime; IMP, imipenem; TET, tetracycline; CHL, chloramphenicol; CLI, clindamycin.

**Table 4:** Antibiotic resistance phenotype, toxin genes, and toxinotype of *C. perfringens* isolated from pork and chicken meat.

No. Sample	Isolate ID	Isolate Source	Resistance Phenotype	Toxin Genes	Toxin Type
1	CC1	Chicken meat	AMP-CTX-TET-CLI	*cpa*	A
2	CC8	Chicken meat	AMP-TET-CHL-CLI	*cpa*	A
3	CC17	Chicken meat	TET	*cpa*	A
4	CC23	Chicken meat	TET-CLI	*cpa*	A
5	CC39	Chicken meat	TET	*cpa*	A
6	CC42	Chicken meat	TET	*cpa*	A
7	CC46	Chicken meat	AMP-CTX-TET-CHL-CLI	*cpa*	A
8	CC50	Chicken meat	TET	*cpa*	A
9	CP2	Pork	TET	*cpa*	A
10	CP5	Pork	AMP-TET-CHL-CLI	*cpa*	A
11	CP8	Pork	TET	*cpa*	A
12	CP11	Pork	AMP-CTX-IPM-TET-CHL-CLI	*cpa*	A
13	CP14	Pork	TET	*cpa*	A
14	CP19	Pork	AMP-CTX-TET-CLI	*cpa*	A
15	CP24	Pork	CHL-CLI	*cpa*	A
16	CP26	Pork	TET	*cpa*	A
17	CP28	Pork	TET	*cpa*	A
18	CP33	Pork	TET	*cpa*	A
19	CP35	Pork	AMP-FOX-CTX-IPM-TET	*cpa*	A
20	CP39	Pork	AMP-TET-CLI	*cpa*	A
21	CP40	Pork	TET-CHL	*cpa*	A
22	CP44	Pork	CLI	*cpa*	A
23	CP47	Pork	TET-CHL	*cpa*	A

## Data Availability

The data that support the findings of this study are available from the corresponding author upon reasonable request.
